# The Arylamidine T-2307 as a Novel Treatment for the Prevention and Eradication of *Candida tropicalis* Biofilms

**DOI:** 10.3390/ijms232416042

**Published:** 2022-12-16

**Authors:** Angela Maione, Alessandra La Pietra, Antonietta Siciliano, Aldo Mileo, Maria De Falco, Elisabetta de Alteriis, Marco Guida, Emilia Galdiero

**Affiliations:** 1 Department of Biology, University of Naples ‘Federico II’, Via Cinthia, 80126 Naples, Italy; 2 National Institute of Biostructures and Biosystems (INBB), 00136 Rome, Italy; 3 Center for Studies on Bioinspired Agro-Environmental Technology (BAT Center), 80055 Portici, Italy

**Keywords:** biofilms, *Candida tropicalis*, invasive fungal infections, in vitro susceptibility, antibiofilm activity, cytokines, *Galleria mellonella*

## Abstract

*Candida tropicalis* is an emerging pathogen with a high mortality rate due to its virulence factors, including biofilm formation, that has important repercussions on the public health system. The ability of *C. tropicalis* to form biofilms, which are potentially more resistant to antifungal drugs and the consequent increasing antimicrobial resistance, highlights an urgent need for the development of novel antifungal. The present study analyzed the antibiofilm capacity of the arylamidine T-2307 on two strains of *Candida tropicalis*. Antimicrobial activity and time-killing assays were performed to evaluate the anticandidal effects of T-2307, the antibiofilm ability on biomass inhibition and eradication was evaluated by the crystal violet (CV) method. Furthermore, in *Galleria mellonella* infected larvae an increased survival after pre—and post- treatment with T-2307 was observed. The MTT test was used to determine the viability of immortalized human prostate epithelial cells (PNT1A) after exposure to different concentrations of T-2307. Levels of interleukin IL-4, IL-8, IL-10 were quantified after Candida infection of PNT1A cells and treatment. Active doses of T-2307 did not affect the viability of PNT1A cells, and drug concentrations of 0.005 or 0.01 µg mL^−1^ inhibited the secretion of inflammatory cytokines. Taken together, these results provide new information on T-2307, indicating this drug as a new and promising alternative therapeutic option for the treatment of Candida infections.

## 1. Introduction

*Candida* spp. are considered the main causative pathogens of septicemia with infections that occur mainly in immunocompromised or hospitalized patients with severe underlying diseases and comorbidities, with a recent prevalence of non-*albicans Candida* spp. One of the most important virulence attributes of such infections is the ability of *Candida* spp. to colonize and form biofilms on several surfaces. Biofilms could also incessantly affect the host immune system and modulate its function and response, often becoming the cause of recurrent and persistent infections that are very difficult to treat [1].

*Candida tropicalis*, an emerging opportunistic pathogen, is nowadays the second or third most common agent of candidemia causing superficial and invasive infections in human populations, especially among neutropenic and oncologic patients also often associated with nosocomial urinary-tract infections. Little is known about the pathogenicity of *C. tropicalis* compared with the more extensively studied *C. albicans* even if *C. tropicalis* express, similarly to *C. albicans*, a wide range of virulence factors such as biofilm formation, yeast-hyphal transition, production of hydrolytic enzymes and enhanced sterol synthesis [2,3]. A major characteristic of *C. tropicalis* infections is the formation of surface attached microbial communities being able to form germ tubes, pseudo hyphae, and hyphae embedded in the extracellular matrix so showing a better ability to form biofilms than *C. albicans*. Accordingly, some researchers reported that biofilm positivity occurred most frequently in the isolates of *C. tropicalis*, that all *C. tropicalis* isolates from fatal infections were intermediate/high biofilm producers so that *C. tropicalis* could be categorized as a strong biofilm producer due to the high biomass production observed in this species [2,4].

Nowadays, the development of mono- and multidrug resistance to antifungals has dramatically increased in several *Candida* species [5]. To overcome these problems, the development of new antifungal agents, high efficacy and low toxicity is the focus for the management of superficial and invasive candidiasis.

In particular, to avoid cross-resistance mechanisms, antifungal agents with novel modes of action need to be urgently developed. The arylamidine T-2307 targets the inhibition of fungal respiratory chain. T-2307, developed by Toyama Chemical Co., Ltd. (Tokyo, Japan), exhibits broad-spectrum antifungal activity against the majority of pathogenic fungi in vitro and in vivo [6,7,8,9], selectively disrupting yeast mitochondrial function, when compared to mammalian cells, which is a different antifungal mechanism among conventional drugs. In a study by Mitsuyama et al. [8], arylamidine T-2307 exhibited potent inhibition against *C. albicans*, including fluconazole- resistant strains, and showed efficacy in the treatment of disseminate candidiasis in a mouse. Furthermore, Wiederhold et al. 2016 [10] demonstrated a strong in vitro and in vivo activity against *C. glabrata*, including echinocandin-resistant isolates, speculating a great efficacy against invasive candidiasis. MICs of T-2307 have been shown, by Abe et al. 2019 [11], to be remarkably low, especially implying that this drug may be effective against ocular candidiasis, even though the ocular permeability of T-2307 is low. That study evaluated the efficacy of T-2307 against *Candida albicans* systemic infections and ocular candidiasis in comparison with that of some conventional anti-fungal agents.

In this work, once ascertained the antifungal activity of T-2307 against a reference and a clinical isolate of *C. tropicalis*, we have investigated the effects of the drug in preventing and eradicating the biofilms formed in vitro by each of the two strains. Further, the expression of some *C. tropicalis* virulence-related genes during biofilm inhibition with the drug were analyzed to provide not yet available information on the effect of T-2307 on *Candida* spp. gene expression. Since *Galleria mellonella* is a well-established model to study in vivo the antimicrobial and also the antibiofilm effect of a drug [12], we also evaluated the antibiofilm activity of T-2307 in pre- and post-infection treatments of the larvae.

The adhesion ability to host epithelial cells is considered an essential virulence attribute of *Candida* spp. [13] because it represents the first step for persistent colonization and establishment of infection. This study, aims to increase the current knowledge about the antifungal potential of T-2307, its effects on human epithelial cells once infected by the two *C. tropicalis* strains were investigated, evaluating (i) the inhibition of adhesion of *Candida* to epithelial cells; (ii) the anti-inflammatory response able to reduce interleukin production, which is known to occur in some type of human cells following *Candida* infection [14,15].

## 2. Results

### 2.1. Minimum Inhibitory Concentration

Microbial strains of *C. tropicalis* DSM 11951 and *C. tropicalis*, as previously identified and characterized, clinical isolate from systemic infection molecularly identified ≥98% and compared with a reference strain *C. tropicalis* EF216862.1, selected on the basis of strong biofilm -forming ability [16], were used. The inhibitory effect of T-2307 on *C. tropicalis* planktonic cells growth, exposed to different concentrations of T-2307, was evaluated by broth dilution. For both strains, the MIC of T-2307 was found to be 0.005 μg mL^−1^ (Table 1). The MIC values for fluconazole found were higher than those reported in various studies, showing that both strains, and especially the clinical isolate, were moderately resistant to fluconazole.

### 2.2. Time-Kill Curves

**Figure 1:**
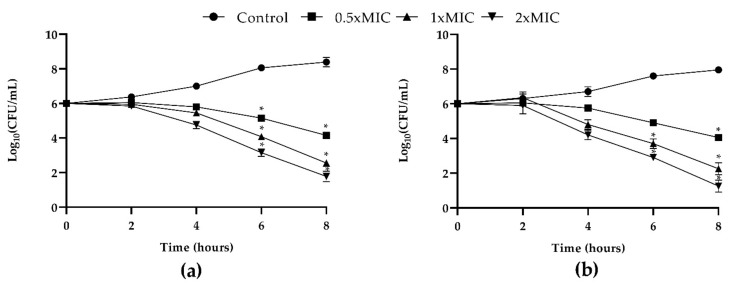
In vitro dose- and time-dependent time-kill assay of *C. tropicalis* strains co-cultured with T-2307: (**a**) growth curves generated using *C. tropicalis* clinical strain cells treated with 0.5× MIC, 1× MIC and 2× MIC of T-2307 and (**b**) growth curves generated using *C. tropicalis* DMS 11951 at concentrations of 0.5× MIC, 1× MIC and 2× MIC with respective controls of strains un-treated. Data expressed as the mean ± standard deviation (log_10_CFU/mL) of three independent experiments * *p* < 0.05 (Dunnett’s test).

The concentration- and time-dependent killing kinetics of T-2307 against both *C. tropicalis* strains were investigated. Results are reported in Figure 1. The untreated fungal control exhibited a progressively increasing growth pattern during incubation. T-2307 exhibited highly significant (*p* < 0.05) antifungal activity from 2 h of co-incubation onward. Subsequently, after 8 h of incubation at all concentrations tested, T-2307 showed a fungicidal action against both strains with about 3, 5 and 6 log reduction for 0.5, 1 and 2× MIC respectively (Figure 1).

### 2.3. Formation of C. tropicalis Biofilms and Evaluation of T-2307 Antibiofilm Activity

**Figure 2:**
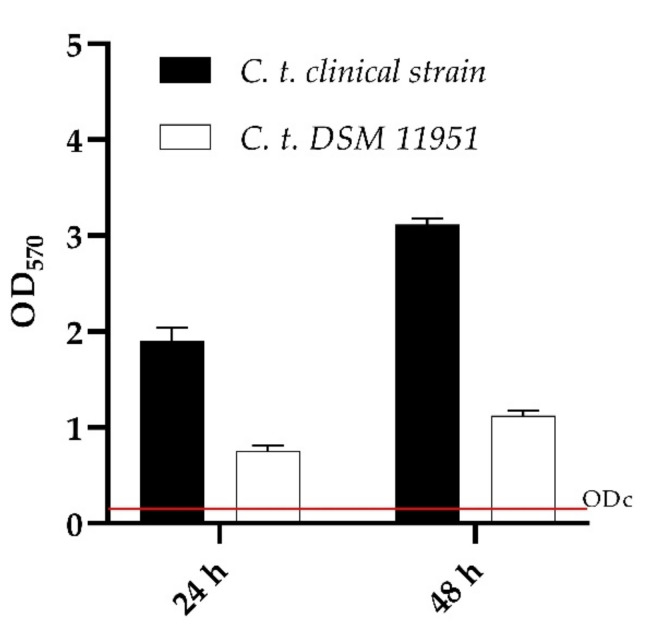
Biofilm formation capacity of *Candida tropicalis* clinical strain and DSM 11951, using the crystal violet staining method. OD cut = mean of negative control with addition of 3 times the SD. Red line represented ODcut.

As shown in Figure 2, both *C. tropicalis* strains had a high capacity to produce biomass demonstrating to be strong biofilm producers according to Stephanovi’c classification.

**Figure 3:**
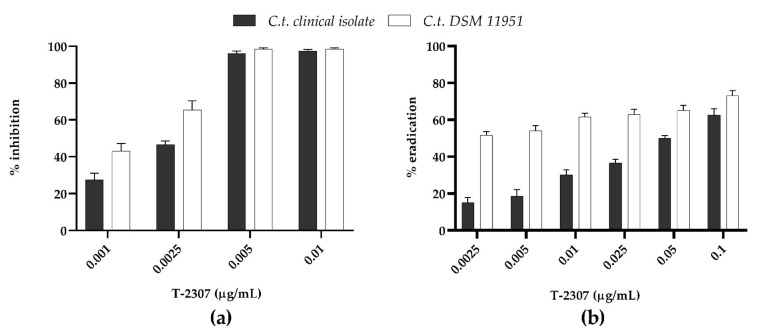
Dose-dependent T-2307 biofilm inhibition (0.25 × MIC, 0.5 × MIC, 1 × MIC and 2 × MIC) and eradication (0.5 × MIC, 1 × MIC and 2 × MIC, 5 × MIC 10 × MIC and 20 × MIC) quantified with crystal violet. (**a**) Ability of T-2307 to inhibit biofilm formation; (**b**) Ability of T-2307 to disrupt mature biofilms.

The activity of T-2307 to inhibit (MBIC) and eradicate (MBEC) the biofilms formed by *C. tropicalis* species is shown in Figure 3. The ability of T-2307 to inhibit biofilm formation (Figure 3a) detected by CV assay, exhibited a dose-dependent inhibition for both *C. tropicalis* DSM11951 and *C. tropicalis* clinical isolate. Interestingly, T-2307 was able to reduce the biofilm formation at sub-inhibitory levels. Indeed, T-2307 was effective at 0.0025 μg mL^−1^ (0.5× MIC) reducing biofilms of 70% and 50% for the DSM11951 strain and the clinical isolate, respectively. The ability of T-2307 to disrupt mature biofilms was also measured (Figure 3b). T-2307 at 0.1 μg mL^−1^ (20× MIC) reduced the total biomass of mature biofilms byabout 70% against both Candida strains. When T-2397 was used at 0.05 μg mL^−1^ (10× MIC) the observed reduction was 60% and 50% for DSM11951 and the clinical strain, respectively relative to the untreated control. In contrast, the total biomass was not reduced by T-2307 at lower concentrations in the case of the clinical isolate, reaching not more than 30% eradication.

### 2.4. ERG11, HWP1, and SAP1,2,3, Genes Expression during Biofilms Inhibition

**Figure 4:**
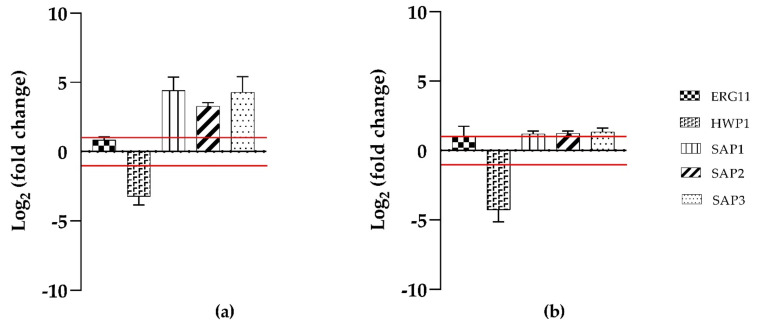
Mean relative mRNA expression levels of ergosterol biosynthesis, hypha specific and virulence response genes of *C. tropicalis* clinical isolate (**a**) and *C. tropicalis* DSM11951 (**b**). Fold changes are calculated according to the formula fold change = 2^−∆∆Ct^ by using Ct (threshold cycle number) generated by the qRT-PCR system. Reported fold changes are the means of three replicate experiments. Red broken lines indicate fold change thresholds of 2 and 0.5 respectively.

To evaluate the effects of T-2307 on the expression of hypha- specific genes (*HWP1*) ergosterol biosynthesis (*ERG11*), and virulence regulator genes (*SAP 1,2,3*), qRT-PCR was applied. Treatment with T-2307 during biofilms inhibition (Figure 4), showed an increase in *ERG11, SAP1, SAP2, SAP3* genes expression and a decrease in *HWP1* gene expression for both strains tested.

### 2.5. In Vivo Anticandidal Efficacy of T-2307

After the showed evidence in vitro of the antibiofilm efficacy of T-2307 on the two strains analyzed, further in vivo efficacy testing was necessary. The larvae of *G. mellonella* are a simple, inexpensive, and quick in vivo screening model to evaluate microbial pathogenicity and host-pathogen interactions. The 50% survival determined in our previous study, for the two *C. tropicalis* strains (1 × 10^6^ CFU/larvae), was confirmed and chosen for further experiments in this study [17].

**Figure 5:**
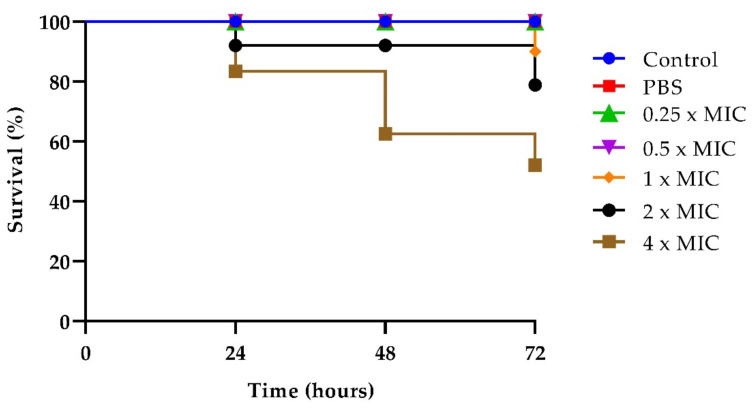
T-2307 toxicity on *G. mellonella* larvae treated with the drug at concentrations of 0.25× MIC, 0.5× MIC, 1× MIC, 2× MIC, 4× MIC.

As reported in Figure 5, no larval mortality was observed in untreated control groups up to 72 h, and also in groups treated with T-2307 at concentrations up to 2 × MIC, indicating that the compound was non-toxic to the larvae at these concentrations. Instead, a significant reduction in survival was observed at 72 h for larvae treated with a 4× MIC concentration of the drug (Figure 5).

**Figure 6:**
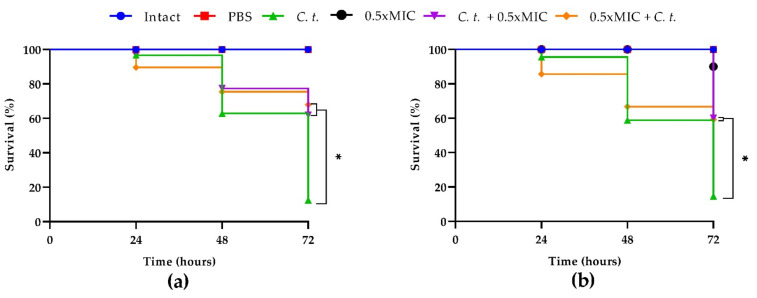
Kaplan–Meier plots of survival curves of *G. mellonella* larvae infected with *C. tropicalis* clinical strain (**a**) and *C. tropicalis* DSM 11951 (**b**). The concentration of candida cells was 1 × 10^6^ CFU/larva. Treatments consisted of phosphate- buffered saline (Control), T-2307 alone (0.5× MIC), T-2307 (0.5× MIC) before or after infection; intact larvae (control). The data are the means of three independent experiments. * *p* < 0.05 (Dunnet’s test).

As regards the anticandidal effect of T2307, *G. mellonella* larvae were treated with the drug at 0.5× MIC before or after the infection with each of the two *Candida* strains (Figure 6a,b).

Both pre- and post-infection treatments showed significantly higher survival rates. In comparison to the infection control group, both pre- and post- treatment groups showed an increase in survival of about 60% at 72 h for the two strains tested.

### 2.6. Cytotoxicity Profile of T-2307 on PNT1A Cells

**Figure 7:**
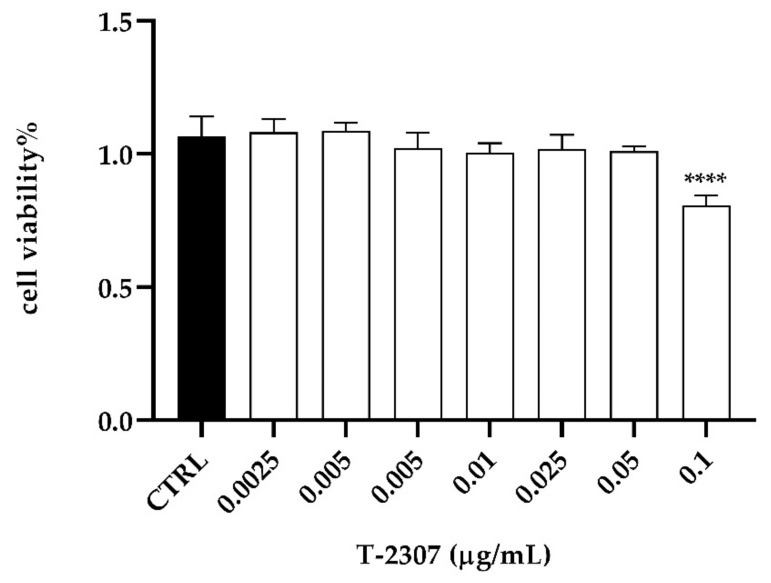
In vitro assessment of cytotoxicity of T-2307 on PNT1A cells detected by MTT assay. **** = *p* < 0.0001 (Tukey’s test).

To assess the cytotoxicity profile of T-2307 on PNT1A cell line, cell viability was assessed after 24 of T-2307 exposure at a concentration ranging between 0.0025–0.1 µg mL^−1^. The results reported in Figure 7 showed that T-2307 did not affect cell viability, which was reduced of 20% only at the highest concentration used. No alteration in cell viability was verified in cellular treatment with the solvent (H_2_O) used to dissolve the compound (CTRL) (Figure 7).

### 2.7. Adhesion Assay

The ability to adhere to epithelial cells contributes to the first step in *Candida* spp. colonization or infection so, we also tested how the T-2307 could affect both *C. tropicalis DSM11951* and *C. tropicalis* clinical isolate adherence to PNT1A cells by measuring the total viable count. After 2 h of infection, the PNT1A cells were washed, and the fungal-adhered cells were enumerated.

**Figure 8:**
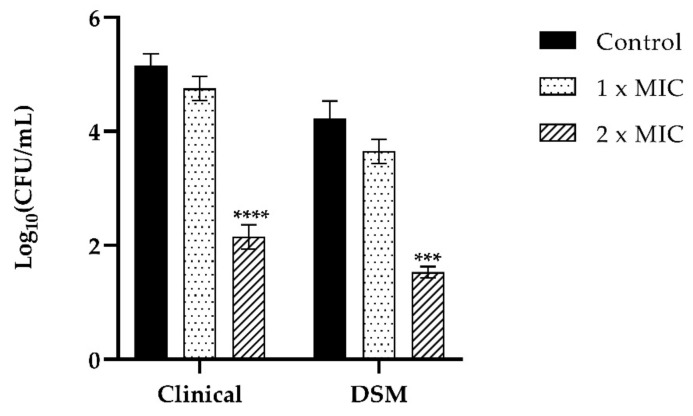
Assessment of anti-adhesion effect of T-2307 (1× MIC, 2× MIC) of *C. tropicalis DSM11951* and *C. tropicalis* clinical isolate on PNT1A cells. Infection of PNT1A cells with the two strains of *C. tropicalis* without treatment was used as a positive control. Data are average of three experiments analyzed in duplicate. **** *p* < 0.0001; *** *p* < 0.001; (Tukey’s test).

Adhesion of *C. tropicalis* clinical strain in the presence of T-2307 at 2× MIC concentration was reduced by 4 log_1_ compared to the control, while 1× MIC concentration of the drug was not potentially inhibitory for fungal adhesion (Figure 8). The same trend was observed for *C. tropicalis* DSM1159 strain as T-2307 inhibited adhesion with a decrease of 2 log compared to the control and no inhibition was observed at 1× MIC concentration (Figure 8).

### 2.8. Anti-Inflammatory Effect of T-2307 on Infected PT1A Cells

**Figure 9:**
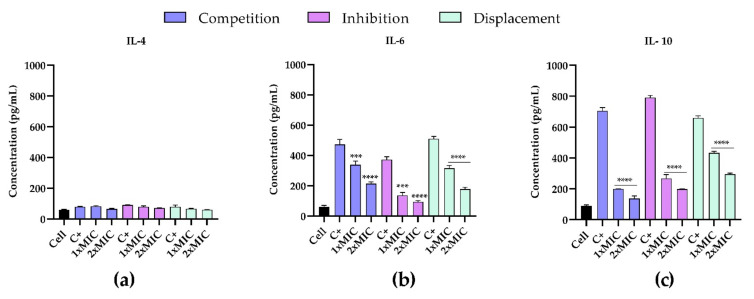
Effects of T-2307 on pro-inflammatory cytokine production in PNT1A cells infected by *C. tropicalis* clinical strain. Detection of IL-4 (panel (**a**)), IL-6 (panel (**b**)) and IL-8 (panel (**c**)). **** = *p* < 0.0001; *** *p* < 0.001; (Tukey’s test).

To investigate the anti-inflammatory effects of T-2307 on infected PNT1A cells, the cell supernatants were analyzed for the presence of pro-inflammatory cytokines IL-4, IL-6 and IL-10 by enzyme-linked immunosorbent assay (ELISA). Analysis of the secretion of such cytokines by PNT1A cells infected with *C. tropicalis* clinical strain and stimulated with T-2307 in three different conditions showed a quite comparable tendency (Figure 9a–c). Treatment with T-2307 significantly suppressed cytokine production. In detail, in all three experiments the competition, inhibition and displacement (Figure 9b,c), there has been a significant increase in the level of IL-6 and IL-10 production in cells infected by the *C. tropicalis* clinical strains when compared with non-treated cells. Treatment with T-2307 at concentrations of 1× MIC and 2× MIC resulted in a decrease in pro-inflammatory cytokines recording a fold-reduction at a maximum of five-fold.

The highest increase was observed in the production of IL-10 (Figure 9c) by infected cells, confirming the important role of IL-10 in regulating the immune response to fungal infections. In all three experiments an evident decrease from 40% to 80% of IL-10 was observed, showing that treatment with the T-2307significantly suppressed this cytokine production. IL-4 levels (Figure 9a) were not detectable in any supernatant from control cells.

## 3. Discussion

*C. tropicalis* has emerged as one of the most important non-albicans Candida species due to its high incidence in systemic candidiasis, its greater resistance to commonly used antifungals, and its ability to produce robust biofilms [18]. These features make *C. tropicalis* infections a challenging problem in medical practice, so that the search for new molecules with antifungal properties become important [2,19]. According to the literature, several mechanisms have been identified as decisive factors for antimicrobial resistance such as the binding of azole to 1,3-glucan at the extracellular matrix (ECM) and its exclusion by azole efflux pumps or mutations in structure or expression of the target protein.

T-2307 is an agent that is currently under investigation and in development for the treatment of invasive fungal infections [20]. Differently from pentamidine, which is structurally similar to T-2307 and known to cause numerous adverse effects and toxicities in animals and humans, when administered systemically, some studies have stated that T-2307 has successfully completed phase I studies with no adverse effects, although these data are not currently available [21]. As well as its pharmacokinetics and formulations for the treatment of patients are unknown to date.

T-2307 is selective for fungi since its mechanism of action causes the collapse of the mitochondrial membrane potential. Indeed, in in vitro studies T-2307 was found to have a greater selectivity for the inhibition of yeast mitochondrial function when compared to that observed against rat and bovine mitochondria [8,22].

In this work we ascertained that the susceptibility of T-2307 for the two *C. tropicalis* strains under examination, was high in accordance with data reported in the literature [21]. Furthermore, our data showed an antibiofilm effect at 24 h against *C. tropicalis* DSM11951 and *C. tropicalis* clinical isolate of T-2307, with significant inhibition of biofilm formation already at a sub-MIC concentration of 0.5× MIC. This effect was corroborated by the result of the expression of the hyphal growth-related gene *HWP1* which appeared downregulated during inhibition.

After 24 h of treatment on preformed biofilms, T-2307 exerted a slightly higher eradication effect on biofilms of *C. tropicalis* DSM11951 compared to clinical strain. *C. tropicalis* is well known for its ability to form strong biofilms, which are difficult to treat especially for the low permeability to antifungal drugs due to the presence of the extracellular polymeric matrix that impedes their penetration [23,24]. Our data confirm that T-2307 promoted biomass reduction in biofilms of both species after 24 h of treatment, but only at high concentrations, suggesting its ineffectiveness in eradicating mature biofilms at sub-MIC doses.

Experiments in the *G. mellonella* model showed the efficacy of T-2307 action as antifungal/antibiofilm also *in vivo*, since the pre- and post- treatment with the drug, provided varying degrees of protection to the larvae, and confirmed that the compound has a selective mode of action for yeast.

Adhesion is the first step in the interaction of a fungal pathogen with the host and in the subsequent possible biofilm formation. Therefore, after ascertaining the non-toxicity of T- 2307 on PNT1A cells, we assessed the anti-infective nature of T-2307 by analyzing *C. tropicalis* adhesion to pre-treated cells, demonstrating that cell counts were reduced from 8% at concentration 1× MIC to 58% at concentration 2× MIC for the clinical strain and from 13.5% at concentration 1× MIC to 64% at concentration 2× MIC for DSM 11951 strain. These data suggest that T-2307 is potential for preventing *C. tropicalis* interaction with epithelial cells.

A new perspective in the field of experimental immunology have confirmed that the host response against fungal organisms requires the coordinated contribution of both innate and adaptive immunity, also indicating the important regulatory role of cytokines [14]. In adaptive response to fungal pathogenesis a number of cytokines are involved, playing a relevant role as regulators in the development of T helper-cell, including IL-1, IL-4, IL-6, IL-8, IL-10 IL-12, IL-15. [25,26]. Understanding the different roles of cytokines is required for the use of antifungal agents in prophylaxis and therapy during fungal infections.

Our evidence showed strong induction in the secretion of the chosen cytokines in PNT1A cells infected by both *Candida* strains and a marked cytokine suppressive effect produced by T-2307, especially in the case of IL-10, demonstrating a strong anti-inflammatory effect associated with the compound. Our data is in accordance with the observation that IL-10 is just produced in response to *C. albicans* hyphae production [27], the reversible switch between the yeast-like to filamentous morphology, an essential Candida virulence trait.

In fact, in the competition experiment when we stimulate the cells simultaneously with T-2307 and the two candida there has been a significant suppression in its level of about 3/5 times compared to the control. The highest suppression of the excessive response to the stimulus has been observed in the inhibition in which we pre-treated the cells with the drug (prevention) and then we infected them but also evident in displacement in which cells are treated with T-2307 after infection, we observed a decrease of approximately 3 or 4 times respectively.

Taken together, the results of this work show the potential antibiofilm activity of T-2307 ability with low toxicity both in vitro and in vivo. Moreover, obtained results provide new information that serves as a basis for future research in the exploration of other possible targets for the antifungal action of this drug.

Although our exposed data are very precious in a context where information about this compound are limited, some limitations should be considered as cytokine dosage and the ecotoxicological data that are unavailable yet. Infact future perspective should go in this direction.

## 4. Materials and Methods

### 4.1. Chemicals

T-2307 was synthesized by Toyama Chemical Co., Ltd. (Tokyo, Japan) and Fluconazole was obtained commercially from Sigma-Aldrich Co. (St. Louis, MO, USA). Stock solutions were obtained by dissolving the powders in DMSO at 50 mg mL^−1^ and 5 mg mL^−1^, respectively. The stock solution of T-2307 was ultrasonicated and the pH was adjusted to 3 with HCl.

### 4.2. Strains and Culture Conditions

*Candida tropicalis* DSM 11951 and *Candida tropicalis* isolate previously recovered from a patient with invasive candidiasis and identified through DNA sequences at multiple loci and biochemical properties in our previous work [16] were selected for the present study. The two fungal strains were stored at –80 °C in Tryptose soya broth (TSB) (OXOID, Basingstoke, UK) 0.1% glucose and with 15% (vol/vol) glycerol. For use, Candida cultures were grown in TSB 0.1% glucose medium for 24 h at room temperature with continuous agitation, washed twice using phosphate- buffered saline (PBS) (OXOID, Basingstoke, UK) and standardized to 10^6^ cells mL^−1^.

### 4.3. Cell Cultures

PNT1A cells (Human prostate cell line established by immortalization of adult prostate epithelial cells) were obtained from the European Collection of Cell Culture (ECACC Salisbury, UK). They were maintained in RPMI 1640 medium (Sigma Aldrich), supplemented with 10% Fetal Bovine Serum (Sigma Aldrich), 2 mM L-glutamine and 100 U/mL penicillin/streptomycin (Sigma Aldrich) in a humidified incubator at 37 °C and 5% CO_2_. After 70% confluency, the cells were harvested, detaching them with Trypsin/EDTA solution (Sigma Aldrich) and cultured into the new flasks. The medium was replaced twice a week.

### 4.4. Minimum Inhibitory Concentration

Antifungal susceptibility testing was performed using the broth microdilution method in 96-well microtiter plates according to the CLSI M27-A3 methods [28] with MICs read after 24 h of incubation at 100% inhibition of growth. Fungal suspensions were prepared in TSB 0.1% glucose (final concentration of 1 × 10^6^ cells mL^−1^) mixed with different concentrations of T-2307 or fluconazole and incubated at 37 °C for 24 h. Fungal growth was determined at 590 nm wavelength with a microplate reader (SYNERGYH4 BioTek, Agilent Technologies,Winooski VT05404 USA). The MIC of the T-2307 was determined as the lowest concentration at which no detectable fungal density was observed. Sterile broth and DMSO were used as negative controls.

### 4.5. Time-Kill Curves

The time–kill kinetics of T-2307 against the two *C. tropicalis* strains were performed according to a previously established protocol [29]. The time–kill kinetics of the T-2307 were assayed at concentrations of 0.005 µg mL^−1^, 0.0025 µg mL^−1^, and 0.01 µg mL^−1^, equivalent to MIC and ½ or 2× MIC. Killing capacity at 0, 2, 4, 6, and 8 h were assessed by plating cells collected and properly diluted, on Tryptose soya agar (TSA) (VWR Chemicals, Leuven, Belgium) plates incubated at 37 °C for 24 h and finally subjected to viable colony counts.

### 4.6. Biofilm Formation

To form *Candida* biofilms in vitro, 100 mL of the yeast cell suspension were added to each well of a 96-well flat-bottomed microplate, incubated for 24 h at 37 °C. Then, non-adherent cells were removed, the wells were washed three times with PBS and residual biofilm biomass was quantified using the crystal violet (CV) staining, as described previously [30,31].

### 4.7. Determination of the Minimum Biofilm-Inhibiting Concentration (MBIC)

Inhibition of biofilm formation was determined in 96-well flat-bottomed plates, adding to100 μL of cell suspension and T-2307 at concentrations of 0.25× MIC, 0.5× MIC, 1× MIC and 2× MIC. The plate was incubated at 37 °C for 24 h. The residual biofilm biomass was quantified by CV staining, as reported above. The percentages of biofilm reduction were calculated as:

% Biofilm reduction = Abs control − Abs sample/Abs control × 100

### 4.8. Determination of the Minimum Biofilm-Eradication Concentration (MBEC)

The biofilm previously formed in 96-well, flat-bottomed plates for 24 h was renewed with fresh medium for another 24 h at 37 °C to obtain a mature 48 h biofilm. Eradication was performed by adding to the mature biofilms T-2307 at concentrations ranging from 0.5× MIC to 20× MIC and incubated at 37 °C for other 24 h. The procedure for revealing and calculating the residual biofilm was the same as described above.

### 4.9. Genomic Analysis

qRT-PCR was used to detect the expression profile of important genes related to biofilm development and infection. The expression of genes involved in hyphal growth and in ergosterol biosynthesis (HWP1, ERG11) and genes related to the production of extracellular hydrolytic enzymes (SAP1, SAP2 and SAP3) were evaluated. Actin (ACT1) was used as a housekeeping gene and the expression of each gene was analyzed and normalized against the ACT1 gene using REST software (Relative Expression Software Tool, Weihen- stephan, Germany, version 1.9.12) based on the Pfaffl method [32,33]. Total RNA was extracted and purified from *C. tropicalis biofilms* grown for 24 h at 37 °C in presence of T-2307 at concentration of ½ MIC using using Direct-zol ™ RNA Miniprep Plus Kit (ZYMO RESEARCH, Irvine, CA, USA) according to the manufacturer’s instruction. For each sample, 1000 ng of total RNA was converted into complementary DNA using an iScript™ cDNA Synthesis kit (Bio-Rad, Milan, Italy) following the manufacturer’s instructions. Primer sequences used for amplification of the specific genes, as described previously [16], were reported in Table 2. qRT-PCR was performed in a AriaMx Real-Time PCR instrument (Agilent Technologies, Inc., Santa Clara, CA, USA); 95 °C for 10 min by 1 cycle, followed by 40 cycles of 15 s at 95 °C and 60 s at 60 °C and 72 °C for 20 s by 1 cycle, 1 cycle for melting curve analysis (from 95 to 60 °C). For the reaction, 1× SensiFAST™ SYBR Green master mix (Meridiana Bioline, UK) was used. Each sample was investigated in triplicate and the transcription levels were calculated using the formula of 2-ΔΔCt method. Fluorescence was measured using Agilent Aria 1.7 software (Agilent Technologies, Inc., Santa Clara, CA, USA).

### 4.10. Galleria mellonella Assays: Toxicity, Infection Rescue

First, a toxicity test was performed by exposing *G. mellonella* larvae to T-2307 at concentration of 4× MIC, 2× MIC, 1× MIC, 0.5× MIC and 0.25× MIC. The larvae, of about 200–250 mg, were injected with aliquots (10 μL) of T-2307 suspensions via the last right pro-leg using a Hamilton syringe (26 gauge) and monitored at 37 °C in a dark environment for 3 d in 3 independent experiments.

The T-2307dose that gave the 50% of larvae survival [34] was used to assess the antifungal activity. The assessment of the in vivo antifungal efficacy of T-2307 at concentration of 0.5× MIC against the two *C. tropicalis* strains was carried out as reported previously [17]. Briefly, a total of 5 groups, each comprising 20 larvae, were formed: group I (*C. tropicalis* DSM 11951 or clinical strain infection with 10^6^ yeast cells per larva); group II (pre-treatment withT-2307 and then infection); group III (infection and after 2 h post-treatment with T-2307); group IV (untreated larvae served as a blank control group, intact larvae); group V (injection with sterile PBS, PBS control). Survival of larvae was recorded every day for 3 d. Larvae were considered dead if they gave no response to slight touch.

### 4.11. In Vitro Cytotoxicity Assay

T-2307 cytotoxicity was assessed by 3-[4,5-dimethylthiazol-2-yl]-3,5 diphenyl tetrazolium bromide (MTT) assay, a colorimetric test that correlates the formazan crystal concentration, created by mitochondrial enzymes with cell viability. Pnt1a cells were seeded in 96- well plate at a density of 5 × 10^3^ cell/well. After starvation, the cells were treated with T-2307 20 × MIC, 10 × MIC, 5 × MIC, 2 × MIC, 1 × MIC, 0.5 × MIC. The drug was directly dissolved into the cell culture medium. Control cells were treated with vehicle (PBS). After 24 h of treatment, MTT solution was added to each well and the cells were incubated for 4 h in a humidified incubator at 37 °C and 5% CO_2_. Then, the medium was gently removed and replaced with DMSO to dissolve the formazan crystals. The absorbance of formazan crystal was measured at 570 nm with a microplate reader.

### 4.12. Adhesion Assay

To study the adhesion ability of the two strains of *C. tropicalis* to PNT1A, once cells were confluent, the below- described procedure was followed. They were treated with T-2307 at a concentration of 0.005 and 0.01 μg mL^−1^ for 2 h at 37 °C. Then, *C. tropicalis* yeast cells (∼6 log CFU/well) were added. After 2 h of incubation and different washings with PBS, adhered *C. tropicalis* cells were recovered by accurately scraping the wells. Serial dilutions were made in PBS, and yeast cells were plated onto Rose Bengal with chloramphenicol (Oxoid) agar plates. After incubation at 37 °C for 48 h, CFU per mL of supernatant was calculated.

### 4.13. Infection of PNT1A Cells with C. tropicalis and T-2307 Treatments

PNT1A were seeded (4 × 10^5^ cells mL^−1^) onto 24-well plates and incubated for 24 h at 37 °C in 5% CO_2_ overnight. The ability of T-2307 to reduce the inflammation in PNT1A cells after infection with *C. tropicalis*, was investigated in three different experimental types: (i) Competitive assay, in which PNT1A cells were infected with *C. tropicalis* DSM 11951 and *C. tropicalis* clinical strain in the presence of T-2307at concentration 0.5× MIC for 2 h at 37 °C; (ii) Inhibition assay, in which cells were treated with the same concentration of T-2307 for 1.5 h and subsequently infected with each strain for 2 h; (iii) Displacement assay in which cells infected with *C. tropicalis* DSM 11951 or *C. tropicalis* clinical strain strains for 2 h were treated with T-2307 at the same concentration) and further incubated for 1.5 h. For all three types of experiments, the plates were centrifuged, and the supernatant was removed and stored at −80 °C for the determination of cytokine levels.

### 4.14. Reduction of Inflammation with Measurement of Cytokine Levels

The supernatant of treated cells was used to measure the levels of selected cytokines produced by Th2 (IL-4, IL-6 and IL-10) using cytokine enzyme-linked immunosorbent assay (ELISA) kits (RayBiotech, Peachtree Corners, GA, USA) by following strictly in accordance the manufacturer’s instructions. The absorbance was quantified at 450 nm using a microplate spectrophotometer (SYNERGYH4 BioTek, Agilent Technologies,Winooski VT05404 USA). Results were expressed as pg/mL.

### 4.15. Statistical Analysis

The data were analyzed using GraphPad Prism Software (version 9.00 for Windows, GraphPad Software, La Jolla, CA, USA, www.graphpad.com, accessed on 20 September 2022). Results were shown as mean values ± standard deviation (SD) of three biological replicates. One-way ANOVA with Dunnett post-test was used for Time-Kill and Survival curves, two-way ANOVA followed by Tukey’s test was used for Cytotoxicity, Adhesion and ELISA assay. Values were considered significant with a *p*-value < 0.05. Kaplan–Meier method was used to plot Survival curves. Expression of gene was analyzed and normalized using REST software (Relative Expression Software Tool, Weihen- stephan, Germany, version 1.9.12).

## Figures and Tables

**Table 1:** Minimum Inhibitory Concentration (MIC) of T-2307 and Fluconazole (FLC) against *C. tropicalis* DSM 11951 and *C. tropicalis*.

	MIC (µg mL^−1^)	
	T-2307	FLC
*C. tropicalis clinical isolate*	0.005	64.0
*C. tropicalis DSM 11951*	0.005	36.0

**Table 2:** Gene-specific primers used for real-time RT-PCR.

Gene Name	Acronym	Primer Name	Sequence (5′->3′)
*Actin*	*actin*	*C. tropicalis_actin_F*	GGCTGGTAGAGACTTGACCAACCATTTG
		*C. tropicalis_actin_R*	GGAGTTGAAAGTGGTTTGGTCAATAC
*Ergosterol biosynthesis*	*ERG11*	*C. tropicalis_ERG11_F*	TTGATTGATTCCTTGTTGGTTA
		*C. tropicalis_ERG11_R*	CATCTTGTAATTGTGGTTGTTC
*Hyphal wall protein 1*	*HWP1*	*C. tropicalis_HWP1_F*	CCCAGAAAGTTCTGTCCCAGT
		*C. tropicalis_HWP1_R*	CCAGCAGGAATTGTTTCCAT
*Secreted aspartyl proteases 1*	*SAP1*	*C. tropicalis_SAP1_F*	TATGACAATGTGCCAGTT
		*C. tropicalis_SAP1_R*	TAAAGCAGTCAAAGTCCC
*Secreted aspartyl proteases 2*	*SAP2*	*C. tropicalis_SAP2_F*	GCTGGTTTCTGTGCTTTG
		*C. tropicalis_SAP2_R*	CCACGTAGGCATGTCTTA
*Secreted aspartyl proteases 3*	*SAP3*	*C. tropicalis_SAP3_F*	ACTTGGATTTCCAGCGAAGA
		*C. tropicalis_SAP3_R*	AGCCCTTCCAATGCCTAAAT

## Data Availability

Not applicable.
